# A prospective observational study comparing outcomes application of low-frequency pulse electrical combined with target-oriented rehabilitation therapy in postoperative nerve function rehabilitation of patients with distal humeral fracture and radial nerve injury

**DOI:** 10.3389/fneur.2024.1370313

**Published:** 2024-04-10

**Authors:** Shaoyan Shi, Xuehai Ou, Xiaolong Du

**Affiliations:** Honghui Hospital, Xi'an Jiaotong University, Xi'an, China

**Keywords:** low-frequency pulse electrical stimulation, goal-oriented rehabilitation, distal humeral condyle fracture with radial nerve injury, nerve function rehabilitation, rehabilitation therapy

## Abstract

**Objective:**

The aim of the present study was to compare the effect of low-frequency pulse electrical stimulation combined with target-oriented rehabilitation therapy and single low-frequency pulse electrical stimulation therapy on postoperative neurological improvement in patients with radial nerve injury and humeral condylar fracture.

**Methods:**

A total of 88 patients with humeral condyle fracture and radial nerve injury admitted to our hospital from April 2019 to January 2022 were randomly divided into a combined group and a control group, with 44 patients in each group. The patients in the combined group received low-frequency pulse electrical stimulation combined with target-oriented rehabilitation therapy, while those in the control group received low-frequency pulse electrical stimulation therapy. The recovery rate of radial nerve function, the recovery of finger extensor and wrist extensor muscle strength, and the occurrence of postoperative complications were evaluated in all patients.

**Results:**

After treatment, the recovery rate in the combined group (77.27%) was higher than that in the control group (50.00%) (*p* < 0.05). There was no significant difference in finger extensor and wrist extensor muscle strength before treatment between the two groups (*p* > 0.05). After treatment, both groups showed improvement compared to before treatment (*p* < 0.05), and the recovery in the combined group was better than that in the control group (*p* < 0.05). There was no significant difference in MCV and amplitude before treatment between the two groups (*p* > 0.05). After treatment, both groups showed improvement compared to before treatment (*p* < 0.05), and the recovery in the combined group was better than that in the control group (*p* < 0.05). The fracture healing time in the combined group was shorter than that in the control group (*p* < 0.05). During the treatment period, there was one case of infection and one case of joint pain in the combined group, with a complication rate of 4.55%. In the control group, there was one case of infection and two cases of joint pain, with a complication rate of 6.82%. There was no significant difference in the complication rate between the two groups (*p* > 0.05). The DHI score in the combined group was better than that in the control group (*p* < 0.05). The ESCA score in the combined group was better than that in the control group (*p* < 0.05).

**Conclusion:**

Low-frequency pulse electrical stimulation combined with target-oriented rehabilitation therapy can promote muscle strength and functional recovery after radial nerve injury, accelerate fracture healing time, and no additional risk of complications.

**Clinical trial registration:**

https://www.researchregistry.com/, researchregistry9461.

## 1 Introduction

Distal humeral condyle fracture often occurs above the medial and lateral condyles of the distal humerus. Clinical symptoms include local swelling, pain, and nerve damage, with radial nerve injury being the most common. Based on the anatomical location of the humerus and radial nerve, the radial nerve runs on the outer side of the distal humerus, above the lateral condyle. When the humerus fractures and displaces severely or produces large bone fragments, it may compress or irritate the nerve, resulting in nerve damage (1). After radial nerve injury, common clinical manifestations include wrist drop deformity, limited finger movement, and sensory impairment in the anatomical snuffbox area. Due to the complex anatomy of the elbow joint and limited space for internal fixation, the overall treatment difficulty is high, and the prognosis is relatively poor. Postoperative complications such as elbow joint stiffness and heterotopic ossification easily occur, severely affecting the patient’s quality of life (2). According to relevant literature, a scientific and reasonable rehabilitation treatment plan is an important measure to prevent elbow joint stiffness. In addition, rehabilitation treatment is beneficial for promoting the recovery of limb function and preventing muscle atrophy and weakness. Low-frequency pulse electrical stimulation therapy is a new non-invasive rehabilitation approach. Currently, low-frequency electrical stimulation has been clinically observed to be beneficial in the treatment of distal humeral fractures combined with radial nerve injury, improving the muscle strength of wrist extensors, thumb extensors, and nerve conduction velocity, ultimately contributing to an improved quality of life for patients (3). Goal-oriented rehabilitation, as a progressive exercise mode, guides patients to achieve phased rehabilitation goals, which can better stimulate patients’ subjective initiative (4).

Low-frequency pulse electrical stimulation therapy, also known as neuromuscular electrical stimulation therapy, has accumulated mature application experience in the medical field due to its advantages of being a non-invasive rehabilitation treatment with high safety and easy operation. This treatment mainly relieves local pain or changes the pathological state by stimulating the affected area with low-frequency electrical currents. In the past few years, the widespread understanding of low-frequency pulse electrical stimulation therapy has been mainly aimed at reducing pain and improving function in different pain situations, and it is the main clinical tool for treating pain (5). The use of low-frequency pulse electrical stimulation therapy in clinical practice can alleviate pain, especially before stretching and therapeutic exercises.

In recent animal and clinical studies, low-frequency pulse electrical stimulation therapy has improved balance, muscle strength, and spastic states (6, 7). Low frequency pulse electrical stimulation therapy effectively relieves muscle fatigue; muscle fatigue is considered an important factor in arbitrary muscle control, posture, and balance (8). The use of low-frequency pulse electrical stimulation therapy effectively reduces knee pain by increasing the motor neuron pool of the quadriceps femoris and triggering isometric quadriceps activity (9). The clinical reports of low-frequency pulse electrical stimulation combined with goal-oriented rehabilitation in the treatment of humeral shaft fractures combined with radial nerve injury are limited. The specific aim of this study was to analyze the effect of two different treatments on postoperative neurological improvement in patients with radial nerve injury and humeral condylar fracture. Therefore, this study investigated the clinical effects of postoperative nerve function rehabilitation in 88 patients with distal humeral condyle fractures combined with radial nerve injury admitted to our hospital from April 2019 to January 2022, aiming to provide reference for selecting appropriate rehabilitation programs in clinical practice. The results are reported as follows.

## 2 Materials and methods

### 2.1 General information

A total of 88 patients with distal humeral fractures complicated by radial nerve injury, admitted to our hospital from April 2019 to January 2022, were selected for this study. Based on a random number table, the patients were divided into a combination group and a control group, with 44 patients in each group. The control group consisted of 29 males and 15 females, aged 32–54 years, with an average age of (42.54 ± 5.27) years. The combination group consisted of 25 males and 19 females, aged 34–55 years, with an average age of (43.17 ± 5.43) years. The general information of the two groups of patients was comparable (*p* > 0.05). The experiments were admitted to the Ethics Committee of the Honghui Hospital, Xi’an Jiaotong University (2021093698). All participants provided informed written consent for their inclusion in the study.

Sample size calculation is based on the hospital sampling survey case–control study method, with an estimated prevalence rate of 5% and a relative error of 20% in the sampling survey. The design efficiency deff is set to 1.5 with reference to other similar large-scale health surveys. A 95% confidence interval is taken, with Za = 1.96 and a data incompleteness rate of 10%. The final calculated sample size ranges from 60 to 100.

Inclusion criteria: (1) Patients diagnosed with a fracture of the humeral condyle confirmed by imaging studies. (2) Patients diagnosed with concurrent radial nerve injury confirmed by electromyography. (3) First time fracture.

Exclusion criteria: (1) Patients with consciousness disorders or mental disorders. (2) Patients with poor compliance and unable to cooperate with subsequent treatment. (3) Patients who withdraw or refuse to continue the study midway. (4) Osteoporosis detected by bone density examination.

### 2.2 Methods

Treatment with low-frequency pulse electrical stimulation was administered to the control group: Prior to low-frequency pulse electrical therapy, thorough communication and discussion of the treatment plan and underlying mechanisms were conducted with the patients and their families to alleviate their adverse psychological state. Once the patients were adequately prepared, they were assisted into the treatment room and placed in a supine position. Treatment was administered using a low-frequency pulse electrical therapy device (Shanghai Yimu Medical Equipment Co., Ltd., registration number 20142090132, model KD-2C), with the mode adjusted to low-frequency pulse electromagnetic therapy. After setting the relevant parameters, the treatment device was applied to the fracture site of the patients for 25 min per session, once a day, until the patients were discharged.

In addition to the treatment received by the control group, the combined group underwent target-oriented rehabilitation therapy. Target-oriented rehabilitation therapy is a progressive rehabilitation exercise model that mainly guides patients to achieve different stages of exercise goals, in order to achieve the maximum rehabilitation effect: (1) In the early stage of the disease, patients and their families were provided with health education on target-oriented rehabilitation programs. Professional rehabilitation therapy teams assessed the patients’ psychological state and physical condition and provided targeted psychological guidance and dietary guidance. (2) During the relatively stable period, passive rehabilitation training was conducted on the patients’ finger joints, wrist joints, and elbow joints, and appropriate physical therapy was applied to the affected limb. Patients were instructed on upper arm muscle contraction training. The patients’ condition was reassessed, and the training intensity was appropriately adjusted based on their recovery status, focusing mainly on wrist joint, elbow joint, and shoulder joint flexion and extension. (3) During the stable period, the focus of training was on abduction and external rotation of the patients’ shoulder, supplemented with appropriate extension and flexion movements to exercise the shoulder joint and muscles.

Both groups of patients received discharge guidance and were followed up through telephone or outpatient visits. Regular hospital visits for reexamination were scheduled.

### 2.3 Observation indicators

(1) Evaluation based on the functional criteria for radial nerve repair after injury developed by the Hand Surgery Society of the Chinese Medical Association (10). The evaluation ranges from 0 to 16, the higher the score, the better the function. In addition, we set the score 9–16 as excellent and good recovery and the score under 8 score as fair and poor recovery. (2) Muscle strength recovery of the finger extensor and wrist extensor muscles is measured using an electromyography device (produced by Zhuhai Maikang Technology Co., Ltd.) before treatment and 6 months after treatment. (3) Motor nerve conduction velocity (MCV) and amplitude: MCV and amplitude of the patients are measured before treatment and 6 months after treatment using an electromyography device. (4) Fracture healing time: Based on the review of imaging data, the time required for the formation of callus, callus volume, and disappearance of the fracture line at the fracture site is recorded for both groups of patients. (5) The incidence of infection, joint pain, and delayed fracture healing during the treatment period is recorded. (6) Duruoz Hand Index (DHI) score: Assessing the level of hand activity in daily life for both groups of patients. (7) Elderly Self-Care Ability (ESCA) score: Evaluating the level of self-care ability in daily life, emotional regulation ability, and social interaction for both groups of patients.

### 2.4 Statistical methods

SPSS 22.0 statistical software package was used for data analysis. Perform Kolmogorov Smirnov test on the data to obtain a normal distribution. Continuous variables were expressed as (mean ± standard deviation) and compared between groups using *t*-test. The categorical variables were expressed as [*n* (%)] and analyzed by Chi-square test for the difference between the groups. A *p* value of less than 0.05 was considered statistically significant.

## 3 Results

### 3.1 Comparison of the excellent and good recovery rates of nerve function between the two groups

The combined group obtained a higher excellent and good recovery rate than the control group (77.27 vs. 50.00%). According to the chi-square test, the combined group has a significant association (*p* < 0.05) with better recovery categorizations, as shown in Table 1.

**Table 1:** Recovery rates of neurological function in two patient groups [*n* (%)].

Group	Number of cases	Excellent and good recovery	Fair and poor recovery
Combined group	44	34 (77.27)	10 (22.73)
Control group	44	22 (50.00)	22 (50.00)
*χ^2^*		7.071	
*p*		0.008	

### 3.2 Comparison of muscle strength recovery between two groups of patients

There was no significant difference in the muscle strength of the finger extensor and wrist extensor muscles before treatment in both groups of patients (*p* > 0.05). After treatment, both groups showed improvement compared to before treatment (*p* < 0.05), with the combination group showing better recovery than the control group (*p* < 0.05). See Table 2.

**Table 2:** Muscle strength recovery in two groups of patients (*x̄* ± *s*).

Grouping	Number of cases	Total extensor muscle		Wrist extensor muscle	
		Before treatment	After treatment	Before treatment	After treatment
Combined group	44	58.64 ± 10.30	220.65 ± 47.67^*^	40.66 ± 23.30	124.33 ± 12.45^*^
Control group	44	59.29 ± 10.35	188.74 ± 43.89^*^	41.67 ± 23.45	101.53 ± 9.86^*^
*t*		0.298	3.303	0.204	9.630
*p*		0.765	0.001	0.838	<0.001

Compared with before treatment in the same group.^*^p < 0.05.

### 3.3 Comparison of MCV and amplitude before and after treatment in two patient groups

There was no significant difference in MCV and amplitude before treatment between the two patient groups (*p* > 0.05). After treatment, both groups showed improvement compared to before treatment (*p* < 0.05), and the combined group showed better recovery than the control group (*p* < 0.05). Refer to Table 3.

**Table 3:** Comparison of MCV and amplitude before and after treatment in two patient groups (*x̄* ± *s*).

Group	Number of cases	MCV (m/s)		Amplitude (mV)	
		Before treatment	After treatment	Before treatment	After treatment
Combination group	44	42.66 ± 4.27	62.58 ± 2.39^*^	10.34 ± 2.41	19.27 ± 3.39^*^
Control group	44	43.71 ± 4.29	50.28 ± 3.41^*^	11.24 ± 2.62	15.23 ± 2.63^*^
*t*		1.150	19.593	1.677	6.245
*p*		0.253	<0.001	0.097	<0.001

Compared with pre-treatment within the same group, ^*^p < 0.05.

### 3.4 Comparison of fracture healing time between the two groups of patients

The fracture healing time in the combination group was shorter than that in the control group (*p* < 0.05). See Table 4.

**Table 4:** Comparison of fracture healing time between the two groups of patients (*x̄* ± *s*, d).

Group	Number of cases	Fracture healing time
Combination group	44	69.42 ± 6.44
Control group	44	78.59 ± 6.39
*t*		6.704
*p*		<0.001

### 3.5 Comparison of complications in two patient groups

During the rehabilitation treatment period, the combined group had one case of infection and one case of joint pain, with a complication incidence rate of 4.55%. The control group had one case of infection and two cases of joint pain, with a complication incidence rate of 6.82%. The frequency of complications in the two groups was similar (*p* > 0.05), see Table 5.

**Table 5:** Complications in two groups of patients [*n* (%)].

Group	Number of cases	Infection	Joint pain	Delayed healing	Total incidence
Combination group	44	1 (2.27)	1 (2.27)	0	2 (4.55)
Control group	44	1 (2.27)	2 (4.55)	0	3 (6.82)
*χ^2^*					0.212
*p*					0.645

### 3.6 Comparison of Dizziness Handicap Inventory scores between two patient groups

The combined group had significantly better DHI scores compared to the control group (*p* < 0.05). Please refer to Table 6.

**Table 6:** Comparison of Dizziness Handicap Inventory (DHI) scores between two patient groups (*x̄* ± *s*_,_ scores).

Group	Number of cases	DHI score
Combination group	44	79.49 ± 7.22
Control group	44	72.07 ± 6.58
*t*		5.038
*p*		<0.001

### 3.7 Comparison of ESCA scores between two patient groups

The ESCA scores in the combined group were significantly higher than those in the control group (*p* < 0.05). Please refer to Table 7.

**Table 7:** Comparison of ESCA scores between two patient groups (*x̄* ± *s*, scores).

Group	Number of cases	ESCA score
Combination group	44	124.66 ± 10.93
Control group	44	100.47 ± 9.85
*t*		10.905
*p*		<0.001

## 4 Discussion

Fractures of the humeral condyle are often caused by indirect violence such as sports injuries and traffic accidents. Under the action of external forces, the fracture site can be displaced forward and downward, leading to radial nerve injury and compression. Additionally, based on the physiological anatomy, the middle and lower third of the humerus closely adheres to the radial nerve. The fractured ends can exert forces such as traction, impingement, and tearing on the radial nerve, ultimately resulting in nerve damage. Furthermore, improper patient handling or inadequate treatment during the transportation of patients with humeral condyle fractures can also cause traction, tearing, or impingement of the nerve, leading to nerve injury (11).

When a fracture of the distal humerus and radial nerve injury occur, potential deformities of the elbow (including valgus deformity) and long-term restricted upper limb mobility often have adverse effects on the physical and mental well-being of the patient (12, 13). Additionally, the treatment options for fractures of the distal humerus and radial nerve injury often carry certain risks, especially invasive and surgical treatments. Due to the small joint cavity volume of the elbow joint and the presence of multiple joints within the cavity, as well as the complexity of the overall structure, there is a significant increase in the risk of complications such as heterotopic ossification and elbow joint stiffness after surgery (14). Timely and correct intervention in neurofunctional rehabilitation can help reduce the risk of complications and promote patient recovery.

In the early stage of fracture, gentle muscle relaxation exercises are performed on the affected limb to promote blood circulation and tissue recovery, as well as to prevent muscle atrophy and joint stiffness. During the relatively stable period, appropriate passive training is conducted to promote muscle strength and joint recovery. In the stable period, the training intensity is appropriately increased based on the patient’s actual condition, promoting the restoration of normal range of motion in the joints (15).

Low-frequency electrical currents are a form of physical energy that, when applied to the treatment site, induce depolarization and hyperpolarization reactions in nerve endings. By activating pre-synaptic inhibition in the spinal cord, blocking ascending conduction, and activating the descending pain inhibition system, it plays a role in local analgesia and accelerated blood circulation (4). In patients with elbow joint immobilization after distal humeral fracture, this treatment can stimulate the damaged radial nerve and muscles, causing passive contractions and thus restoring the function of the damaged nerves and muscles. At the same time, low-frequency electrical stimulation of the nerve terminals promotes smoother lymphatic and blood circulation in the painful area, helping to alleviate pain. Furthermore, electrical stimulation promotes the proliferation of phagocytes and accelerates the decomposition of distal axons in nerve injuries, providing a favorable environment for proximal nerve regeneration. Multiple clinical reports have confirmed the efficacy of low-frequency pulse electrical stimulation in the treatment of distal humeral fractures and radial nerve injuries (16).

The results of this study suggest that the combined group had a higher rate of excellent neural functional recovery compared to the control group. This is because electrical stimulation improves blood circulation in the affected limb, thereby accelerating the exchange of nutrients required for nerve repair. Low-frequency pulse electrical stimulation is beneficial for the proliferation and differentiation of bone cells, thus accelerating bone healing (17). Targeted rehabilitation therapy for localized swelling and bleeding after fracture promotes soft tissue recovery and relieves pain at the fracture site. Additionally, promoting fracture healing helps in the formation of callus and enhances bone density to a certain extent. Targeted rehabilitation therapy, through passive training of the affected limb, helps reduce the risk of joint stiffness. Furthermore, in both groups of patients, although there was no significant difference in muscle strength of the fingers and wrist extensor muscles before treatment, both groups showed improvement in muscle strength after treatment, with the combined group showing better recovery than the control group. Similar to the results of previous studies by Wang Lina et al. (18). Furthermore, the incidence of complications in the research group patients was lower than that in the control group, although this difference was not statistically significant. Low-frequency pulse therapy can stimulate neuromuscular activity, leading to muscle contraction, promoting arterial blood supply, as well as venous and lymphatic drainage, improving local nutrient metabolism, and reducing edema. It can also increase muscle tension, preventing or delaying muscle atrophy. By rhythmically stimulating neuromuscular activity with low-frequency and small current (<15 mA), the risk of adhesion between muscle fibers and fascia, as well as between muscle bundles due to injury or inflammation, can be reduced, maintaining muscle elasticity and preventing muscle spasms. On the other hand, electrical stimulation can promote the regeneration of damaged nerve fibers (19). Low-frequency pulse electrical stimulation can improve blood circulation and metabolism at the fracture site, while targeted rehabilitation therapy, based on passive training of the affected limb, further promotes blood circulation. Previous literature (4, 5) has reported successful cases using this treatment protocol, and relevant reports have demonstrated its effectiveness and safety. In addition, both treatment modalities have a similar safety profile.

Wang et al. (18) conducted a clinical controlled study on 86 patients with humeral shaft fractures combined with radial nerve injury who underwent open reduction and internal fixation surgery. They found that patients who received combined low-frequency pulse electrical therapy had a higher rate of excellent neurological function after 6 months of treatment. In addition, based on clinical indicators such as electromyography (EMG) indicators of wrist extensor muscle strength recovery, motor nerve conduction velocity (MCV), waveform, and fracture healing time, patients in the combined treatment group performed better than the control group during the same period, and these differences were statistically significant (*p* < 0.05). Another clinical report (20) on the combined use of low-frequency electrical stimulation and action observation therapy in patients with upper limb dysfunction after stroke showed that compared to the control group receiving conventional rehabilitation treatment, the experimental group receiving combined low-frequency electrical stimulation therapy demonstrated better motor function scores (FMA) and activities of daily living scores (MBI) after the same treatment period. Additionally, the experimental group also achieved better results in hand function scores (UEFT).

Although several clinical studies have demonstrated the significant efficacy of low-frequency pulsed electrical stimulation in the treatment of humeral fractures with associated radial nerve injury, the underlying treatment mechanism remains incompletely understood. Future research should focus on elucidating the mechanisms by which low-frequency pulsed electrical stimulation affects bone cells, as well as its effects on plasma ions such as calcium, magnesium, and phosphorus in the human body. The limitation of this study is the lack of long-term functional follow-up data for the combined group. Whether the combined group is still better than the control group in long-term observation will be explored. In the future, the sample size will be expanded and multicenter, long-term follow-up experiments will be conducted.

Low-frequency pulse electrical therapy can alleviate patient pain, promote neurological recovery, and facilitate fracture healing, yielding significant clinical effects. Goal-oriented rehabilitation therapy also overcomes the shortcomings of insufficient patient subjective involvement in previous conventional rehabilitation treatments. The combined use of these two rehabilitation approaches can to a certain extent maintain the physical and mental well-being of patients, allowing them to better return to their daily work and life after completing the rehabilitation training.

## Data availability statement

The original contributions presented in the study are included in the article/supplementary material, further inquiries can be directed to the corresponding author.

## Ethics statement

The experiments were admitted to the Ethics Committee of the Honghui Hospital, Xi'an Jiaotong University (2021093698). The studies were conducted in accordance with the local legislation and institutional requirements. The participants provided their written informed consent to participate in this study.

## Author contributions

SS: Conceptualization, Data curation, Formal analysis, Investigation, Resources, Software, Supervision, Validation, Visualization, Writing – original draft, Writing – review & editing. XO: Conceptualization, Data curation, Formal analysis, Investigation, Methodology, Project administration, Resources, Software, Supervision, Writing – original draft, Writing – review & editing. XD: Conceptualization, Data curation, Formal analysis, Investigation, Methodology, Project administration, Resources, Software, Supervision, Validation, Visualization, Writing – original draft, Writing – review & editing.

## References

[1] Entezari V Olson JJ Vallier HA. Predictors of traumatic nerve injury and nerve recovery following humeral shaft fractures: short title: traumatic nerve palsy after humeral shaft fracture. J Shoulder Elb Surg. (2021) 30:2711–9. doi: 10.1016/j.jse.2021.04.025 33964428

[2] Li RB Fu G Lin FF. Prospects for the diagnosis and treatment of post-traumatic elbow stiffness. Chin J Shoulder Elbow Surg. (2021) 9:196–9. doi: 10.3877/cma.j.issn.2095-5790.2021.03.002

[3] Nakano H Naraba H Hashimoto H Mochizuki M Takahashi Y Sonoo T. Novel protocol combining physical and nutrition therapies, intensive goal-directed rehabilitation with electrical muscle stimulation and nutrition (IGREEN) care bundle. Crit Care. (2021) 25:415. doi: 10.1186/s13054-021-03827-8, PMID: 34863251 PMC8645074

[4] Liu M Li S Wang HL Li YM Zhao Y Shi HW. Effect of low-frequency electrical stimulation on the treatment of humeral fractures combined with radial nerve injury. J Hebei Med Univ. (2020) 41:58–60. doi: 10.3969/j.issn.1007-3205.2020.01.014

[5] Karasuno H Ogihara H Morishita K Yokoi Y Fujiwara T Ogoma Y. The combined effects of transcutaneous electrical nerve stimulation (TENS) and stretching on muscle hardness and pressure pain threshold. J Phys Ther Sci. (2016) 28:1124–30. doi: 10.1589/jpts.28.1124, PMID: 27190439 PMC4868199

[6] Cho HY Kim EH Kim B Lee GE Hahm SC Lee GC. Effects of repetitive high frequency transcutaneous electrical nerve stimulation (HF-TENS) on spasticity and motor function following spinal cord injury in rats. J Phys Ther Sci. (2012) 24:133–7. doi: 10.1589/jpts.24.133

[7] Cho HY Sung in T Hun Cho K Ho Song C. A single trial of transcutaneous electrical nerve stimulation (TENS) improves spasticity and balance in patients with chronic stroke. Tohoku J Exp Med. (2013) 229:187–93. doi: 10.1620/tjem.229.187, PMID: 23419328

[8] Cho HY Lee SH in TS Lee KJ Song CH. Effects of transcutaneous electrical nerve stimulation (TENS) on changes in postural balance and muscle contraction following muscle fatigue. J Phys Ther Sci. (2011) 23:899–903. doi: 10.1589/jpts.23.899

[9] Hopkins J Ingersoll CD Edwards J Klootwyk TE. Cryotherapy and transcutaneous electric neuromuscular stimulation decrease arthrogenic muscle inhibition of the vastus medialis after knee joint effusion. J Athl Train. (2002) 37:25–31. doi: 10.1055/s-2002-19375 PMID: 12937440 PMC164304

[10] Pan DD Gu YD Shi D Shou KS. Trial application standards for partial functional evaluation of the upper limbs by the hand surgery Society of the Chinese Medical Association. Chin J Hand Surg. (2000) 16:4–9.

[11] Dolan RT Giele HP. Radial nerve palsies associated with pediatric supracondylar humeral fractures: a caution in the interpretation of neurophysiological studies. J Pediatr Orthop B. (2020) 29:162–32. doi: 10.1097/BPB.0000000000000680 31567895

[12] Isabelle S Werdie SV Lizelle F. Psychological resilience and vulnerability as mediators between adverse life events and fatigue, motor dysfunction, and paresthesia in multiple sclerosis. Psychosom Med. (2020) 82:138–46. doi: 10.1097/PSY.0000000000000770, PMID: 31860531

[13] Mete G Orhan B Tahir Ö Erpala F Eren MB Zengin EC. Does rotational deformity cause poor outcomes after pediatric supracondylar humerus fractures? Turk J Trauma Emerg Surg. (2023) 29:811–7. doi: 10.14744/tjtes.2023.43413, PMID: 37409923 PMC10405028

[14] Han J Hong Y. The impact of evidence-based clinical nursing pathway combined with goal-directed repetitive functional training on functional recovery, rehabilitation motivation, and quality of life of postoperative patients with cerebral hemorrhage. Clin Med Res Pract. (2023) 8:126–8. doi: 10.19347/j.cnki.2096-1413.202304038

[15] Dong Y Han YJ. Therapeutic effects of low-frequency pulse electrical stimulation combined with limb rehabilitation exercise on post-stroke hemiplegic patients. Lab Med Clin Pract. (2022) 19:2707–10.

[16] Xie X Wei JH Liu S Li C Liao B Wang YJ. Application and efficacy of low-frequency pulse electrical stimulation combined with swallowing training in the treatment of swallowing disorders after acute ischemic stroke. West China Med J. (2023) 35:875–80. doi: 10.3969/j.issn.1672-3511.2023.06.017

[17] Li YJ. The effects of low-frequency electrical stimulation combined with rehabilitation training on the treatment of stroke and its impact on motor ability. J Chronic Dis. (2021) 22:894–5.

[18] Wang LN Shi D Du LF Tan Y Zhang LJ. Application of low-frequency pulse electrical stimulation combined with target-oriented rehabilitation pathway in the rehabilitation of humeral shaft fractures combined with radial nerve injury. Hainan Med J. (2023) 34:787–91. doi: 10.3969/j.issn.1003-6350.2023.06.006

[19] Andrew H Kishore M Hosalkar H. The treatment of pediatric supracondylar humerus fractures. J Am Acad Orthop Surg. (2012) 20:7–320. doi: 10.5435/JAAOS-20-05-328 22553104

[20] Wang YL. Application and observation of low-frequency electrical stimulation combined with action observation therapy in patients with upper limb dysfunction after stroke. Chin J Prescrip Drugs. (2023) 21:182–5.

